# Combined effects of reflexology massage and respiratory relaxation on pain following chest tube removal in heart surgery patients

**DOI:** 10.1186/s13019-024-03254-0

**Published:** 2025-01-10

**Authors:** Zainab Bahramian, Majid Kazemi, Reza Vazirinejad, Hadi Hasani

**Affiliations:** 1 Department of Medical Surgical Nursing, Faculty of Nursing and Midwifery, Rafsanjan University of Medical Sciences, Rafsanjan, Iran; 2 Department of Medical Surgical Nursing, Faculty of Nursing and Midwifery, Non- Communicable Disease Research Center, Rafsanjan University of Medical Sciences, Rafsanjan, Iran; 3 Faculty of Nursing, Nurse Street, Rafsanjan, 7718796755 Iran; 4 Department of Epidemiology and Biostatistics, School of Health, Social Determinants of Health Research Center, Rafsanjan University of Medical Sciences, Rafsanjan, Iran; 5 Department of Nursing, School of Nursing and Midwifery, Shahroud University of Medical Sciences, Shahroud, Iran

**Keywords:** Reflexology massage, Respiratory relaxation, Chest tube removal, Heart surgery

## Abstract

**Background and aim:**

Removing the chest tube in cardiac patients after surgery is one of the worst experiences of hospitalization in the intensive care units. Various pharmacological and non-pharmacological methods are available to control pain in these patients. This study aimed to investigate the combined effect of reflexology massage and respiratory relaxation on pain following chest tube removal in cardiac surgery patients of Shahid Beheshti Hospital in Shiraz, Iran, in 2023.

**Methods:**

This was a double-blind randomized clinical trial performed on 140 patients who underwent heart surgery and had a chest tube in Shiraz, Iran. The samples were randomly divided into four groups: 1- control group, 2- respiratory relaxation group, 3- foot reflex massage group, and 4- a combination of respiratory relaxation and reflexology massage. To collect data, two demographic questionnaires, and a visual analog scale were used.

**Results:**

The participants of the four groups were not meaningfully different in terms of age, BMI, duration of surgical operation, gender, job, education, place of residency, number of chest tubes, history of operation (*P* = 0.99, 0.31, 0.06, 0.81, 0.97, 0.96, 0.17, 0.10, 0.89 respectively). The mean scores of pain intensity during chest tube removal, and 15 min after chest tube removal were not statistically different among the four groups of study (*P* = 0.15, 0.54 respectively); However, just after chest tube removal, the mean scores of pain intensity differed meaningfully among four groups (*P* = 0.008).

**Conclusion:**

The results showed that reflexology massage and respiratory relaxation both reduce pain immediately after chest tube removal in heart surgery patients. Also, the combination of these two techniques was more effective in reducing patients’ average pain.

## Introduction

The prevalence of cardiovascular diseases has significantly increased over the past two centuries, making it the leading cause of death, disability, and also the cause of reduced quality of life globally [1, 2].

Almost half of adults in the United States have at least one type of heart disease. This disease affects people of any age, gender, ethnicity, and social and economic levels [3, 4]. According to epidemiological reports, these diseases were the most common cause of death in Europe until 2022 [5]. In Iran, these diseases have become the first main cause of death, which has led to 46% of all deaths and 20–23% of the disease burden [6]. According to studies, the rate of cardiovascular diseases in Iran has increased in recent years by 20–45% according to different studies [7].

In open heart operations, to facilitate the expansion of the lungs and to open a way out for discharging secretions and fluids, at least one chest tube is required [8]. Removing this chest tube can cause pain due to the adhesion to the surrounding tissues; also, it can cause the activation of skin, somatic, and visceral nervous fibers [9]; based on previous studies, patients face moderate to severe pain when removing the chest tube; which this problem is poorly controlled in most of the cases [10, 11]. Removing the chest tube is considered a painful and uncomfortable experience for the patient; described as one of the worst experiences of hospitalization in the intensive care unit [12]. Therefore, the lack of pain control can have negative effects on people’s health [13]; there is still no clear protocol that its use is considered routine care for the management of pain caused by this technique. Nurses should have an evident-based plan to relieve possible pain before starting any painful procedure; especially for pain following chest tube removal [14, 15].

Successful pain management depends on assessment, adequate pharmacologic and nonpharmacologic interventions, and assessment of patient response. There are different ways to control the pain caused by chest tube removal. The use of systematic treatment methods (narcotic and non-narcotic drugs), local anesthesia methods (epidural analgesia, etc.) and complementary medicine methods (massage therapy, sedation, and acupuncture) are among the various techniques. pain control [11, 16]. Currently, painkillers, especially narcotic drugs, are used in special care units to relieve pain after open heart and coronary artery surgery. Although the above-mentioned drugs are considered the most effective means available to reduce the pain for these patients; they have different side effects including respiratory distress, hemodynamic changes, nausea and vomiting, and drowsiness. Also; the degree of drug response in different patients may vary according to lots of clinical conditions like chronic diseases, age, addiction to smoke or opioids, etc. Therefore, it is better to consider a non-pharmacological method alongside these drugs [17].

Relaxation is defined as the absence of physical, mental, and emotional tension and can help in managing pain. Physiologically, relaxation leads to a reduction of the sympathetic response to pain, which leads to a decrease in oxygen consumption, blood pressure, heart rate, and respiration. Psychologically, distraction is an internal component of relaxation and affects pain management by reducing cognitive awareness of pain [18].

There are various methods of relaxation, one of which is slow and deep breathing exercises. According to some studies, migraine pain and pain after surgery can be reduced with the help of this exercise [11, 19]. It has been also reported that respiratory relaxation is an effective technique for managing pain intensity caused by chest tube removal in patients after open heart surgery [11].

In addition, the use of other complementary medicine methods such as massage therapy has gained popularity in recent years. Massage therapy by manipulating soft tissues can bring the metabolic balance of tissues and increase the blood circulation in the tissue [20]. Eunice Lingham, the founder of foot reflexology, drew a map of the feet in the 1930s and claimed that massaging some points of the feet using a unique technique can increase the blood flow of the relevant organs to each of those points on the foot [21, 22].

Reflexology massage of the upper half of the left foot can affect cardiovascular functioning. It can increase blood supply to organs, increase parasympathetic stimulation, and reduce sympathetic stimulation and tension. Also, it has been said that this intervention may stimulate the parasympathetic nervous system through the hypothalamus which causes a decrease in metabolism, heart rate, blood pressure, breathing rate, oxygen consumption, and pain [23–27].

Reflexology, which involves stimulating specific points on the feet, is believed to release blocked energy and reduce pain [20, 28]. This method, along with massage therapy, can release endorphins and enkephalins, providing comfort and relaxation [29]. Previous studies have shown that foot reflexology massage can effectively reduce infant colic pain, women’s back pain, anxiety, and cardiac dysrhythmia in patients undergoing catheterization and pain caused by sternotomy in patients undergoing heart surgery [29, 30]. Babatbar et al. reported that foot reflexology massage during chest tube removal reduced heart rate and breathing rate back to normal values [31]. Babajani et al. also reported that reflexology foot massage is a useful nursing intervention in the chest tube removal procedure after open heart surgery [20].

Despite its benefits, the use of reflexology and other complementary methods in clinical practice is limited due to the existence of doubts regarding the effectiveness of complementary medicine in medical practice may be limited. The need for more evidence-based research to support the effectiveness of these non-pharmacological methods made us design a study in this regard. Also, the combination of these non-pharmacological methods to reduce pain may have double effects compared to the separate use of each method. Therefore, this study aimed to determine the combined effects of two methods of reflexology massage and respiratory relaxation on the intensity of pain following chest removal in Shahid Beheshti Hospital in Shiraz, Iran in 2023.

## Methods

### Study design and participants

This was a double-blinded randomized clinical trial in which 140 patients hospitalized in the heart surgery ward in Shahid Beheshti Hospital in Shiraz, Iran in 2023 assessed.

The patients were selected with a randomized complete block design (blocks of four). The sampling process continued until the sample size reached 140. Random block sampling was used to have an equal number of participants in each of the four groups. For this, the researcher had to randomly select four patients in a row using a simple coin toss method. The simple coin toss method was used because there was no list of the patients at the beginning of the study. Each patient entered the open-heart surgery ICU after heart surgery if he had a chest tube, was decided whether to be in the study or not using the coin toss method. After randomly selecting four patients, the researcher then randomly tagged one of the four patients as “A” and then the next patients were tagged as B, C, and D according to the timeline they were randomly selected. Then, the letters A, B, C, and, D were randomly assigned to one of the four groups of study. For this, we created random numbers in the Microsoft Excel program. The process continued multiple times until we reached the sample size needed. In this way, we could ensure that we have four equal groups each and have selected and assigned patients randomly among the groups of study.

For the blinding purpose of the study, a nurse who worked at the ICU had to record the pain intensity and the data, so the possible researcher bias did not impact the results. Also, the patients knew the concept of the study but did not know that there were whether in single relaxation groups or mixed methods groups and did not know that other patients had different methods used for them. Also, the pain intensity data for the control group was routinely checked by the nurses, and along with the fact that they had no information that some of the patients were in the intervention groups and received complementary and alternative therapies, therefore, their data were also considered blinded. Therefore, the study could have their initial aim to use the double-blinded design.

The required sample size was calculated 35 for each of the four groups; using the below formula with an alpha error of 5% and the beta error of 10%. In this formula, the difference between groups is considered 2, and the standard deviation is considered 1.96; according to a previous study [20].

$$n=\frac{2(z_{1}-\frac{a}{2}+z_{1-\beta})^{2}\quad \sigma^{2}}{\text{d}^{2}}$$

### Inclusion criteria

The inclusion criteria were: 1- willingness to participate in the study, 2- being at least 18 years old, 3- patients who only had a pleural chest tube, 4- awareness of time, place, and persons, and 5- ability to understand the visual scale of pain, 6- absence of severe vision and hearing impairment, 7- having no neuropathy and sensory disorders caused by diabetes 8- not being addicted to drugs and alcohol.

### Exclusion criteria

The exclusion criteria were: 1- being a reluctance to continue participating in the study, 2- being connected to the ventilator, 3- receiving narcotic pain medication less than 4 h before the intervention.

### Instruments

To collect information, the demographic questionnaire, and a visual analog (VAS) scale were used. The demographic information questionnaire was researcher-made; and it included information such as: age, gender, marital status, employment status, education, place of residence, BMI, type of operation, duration of operation, duration of chest tube, number of chest tubes, history of drug addiction, history of neuropathy, history of heart surgery and history of having a chest tube. These variables were included in a demographic questionnaire using previous studies [32–36].

The visual analog scale (VAS) for assessing the pain was used which is a ten-point ruler to determine pain as zero (no pain), 1–3 (mild pain), 4–6 (moderate pain), 7–9 (severe pain), and 10 (the highest pain). This scale was first introduced by Hayes and Patterson (1921) [37]. The validity and reliability of this scale was proven in previous studies [11, 38–42]. Also, in Iran, Rezvani et al. reported that the reliability of this scale is acceptable (*r* = 0.88) [39].

### Intervention

The intervention was started on the first day after the surgery, the researcher selected the participants randomly and based on inclusion criteria. After the baseline assessment, the participants were randomly divided into four groups using a randomized complete block design.

The four groups in our study were: 1- Control group, in this group chest tube removal was done by the routine method. 2- Respiratory relaxation group which was performed using breathing methods before the chest tube was removed. 3- Foot reflexology group; 4- Group of combination of respiratory relaxation and reflex massage, in this group, to control the pain during chest tube removal, before the procedure, first respiratory relaxation and then reflex foot massage was performed.

During the chest tube removal, the patients were in a semi-sitting position, and a pillow was placed under the patient’s head and knees. In the respiratory relaxation groups, to relax the patients, 5 min before the chest tube was removed, slow and deep breathing exercises were performed; In this way, while closing his eyes, the patient took a slow and deep breath through his nose and slowly exhaled with his pursed lips. At the end of 5 min, the chest tube was removed by a nurse [11].

In foot reflexology groups, the center of one-third of the sole of the left foot patient at the anterior side, between the arch of the foot and the toes, a knuckle under the second toe and thumb (Ball of the foot) massaged. The massage was done moments before the chest tube was removed. The massage was performed for ten minutes circularly. (Like crushing a sugar cube with the thumb). The massage was deep and did not cause discomfort and pain to the patient [20, 28].

In the control group, the tube was removed according to the routine procedure of the department. A nurse removed the chest tubes of all patients within one or two minutes in all groups. The pain intensity was measured by a nurse of the ward, who was unaware of the grouping of patients and had received the necessary training on how to measure pain intensity using an analog visual scale. The pain intensity scores were gathered before and after chest tube removal in all the participants.

### Data analysis

After collecting the data, SPSS version 24 software was used to analyze the data. The descriptive statistics (including mean, standard deviation, and frequency) and inferential statistics including chi-square, and one-way ANOVA tests, were analyzed by the above-mentioned software, and then the significance level of the P-value was assessed.

### Ethical considerations

The study protocol was approved by the ethics committee of Rafsanjan University of Medical Sciences (Code: IR.RUMS.REC.1401.236). Also, it is registered in the Iranian Registry of Clinical Trials (IRCT) database (Code: IRCT20131228015965N22).

Before the study, written informed consent to participate in the study was obtained from the participants. Also, the information entered into SPSS software anonymously and the patient’s information was considered highly confidential.

### Findings

In the present study, 140 participants entered the four groups of the study (35 patients in each group); who all continued the study to the end.

The participants of the four groups were not meaningfully different in terms of age, BMI, and duration of surgical operation, according to the One-Way ANOVA test (*P* = 0.99, 0.31, 0.06 respectively). Also, the Chi-square test showed no significant difference between groups in terms of gender, job, education, place of residency, number of chest tubes, and history of operation (*P* = 0.81, 0.97, 0.96, 0.17, 0.10, 0.89 respectively). (Table 1)

**Table 1:** The characteristics of participants among four groups

Variables		Control Group	Respiratory relaxation	Reflexology massage	Combination of respiratory relaxation and reflexology	*P* value
Age (Mean ± SD)		60.57 ± 9.90	61.05 ± 8.21	61.17 ± 9.59	60.74 ± 9.58	0.99 *
BMI (Mean ± SD)		25.70 ± 3.44	24.23 ± 3.55	24.97 ± 2.85	33.12 ± 44.33	0.31*
Surgical operation duration (hour) (Mean ± SD)		5.42 ± 0.55	5.45 ± 0.50	5.65 ± 0.48	5.68 ± 0.47	0.06*
Gender (n. (%))	Female	12 (34.3%)	8 (22.9%)	11 (31.5%)	11 (31.5%)	0.81**
	Male	23 (65.7%)	27 (77.1%)	24 (68.5%)	24 (68.5%)	
Job (n. (%))	Employed	11 (31.5%)	13 (37%)	13 (37%)	13 (37%)	0.97**
	Retired	11 (31.5%)	13 (37%)	11 (31.5%)	11 (31.5%)	
	Housewife	13 (37%)	9 (26%)	11 (31.5%)	11 (31.5%)	
Education (n. (%))	Illiterate	12 (34.3%)	11 (31.5%)	9 (25.7%)	8 (22.9%)	0.96**
	Elementary school	11 (31.5%)	12 (34.3%)	13 (37%)	13 (37%)	
	Middle school	4 (11.4%)	4 (11.4%)	7 (20%)	6 (17.1%)	
	High school	2 (5.7%)	1 (2.9%)	1 (2.9%)	1 (2.9%)	
	Diploma	1 (2.9%)	5 (14.2%)	2 (5.7%)	3 (8.7%)	
	Bachelor or higher	5 (14.2%)	2 (5.7%)	3 (8.7%)	4 (11.4%)	
Place of residency (n. (%))	Urban	30 (85.8%)	23 (65.7%)	23 (65.7%)	23 (65.7%)	0.17**
	Rural	5 (14.2%)	12 (34.3%)	12 (34.3%)	12 (34.3%)	
Number of chest tubes (n. (%))	2	29 (82.9%)	29 (82.9%)	31 (88.6%)	23 (65.7%)	0.10**
	3	6 (17.1%)	6 (17.1%)	4 (11.4%)	12 (34.3%)	
History of operation (n. (%))	Yes	12 (34.3%)	9 (25.7%)	10 (28.5%)	9 (25.7%)	0.89**
	No	23 (65.7%)	26 (74.3%)	25 (71.5%)	26 (74.3%)	

* One Way ANOVA Test

** chi-square

For assessing the intensity of pain in patients; the One-Way ANOVA test was used. According to the results from this test, the mean scores of pain intensity during chest tube removal, and 15 min after chest tube removal were not statistically different among the four groups of study (*P* = 0.15, 0.54 respectively); However, just after chest tube removal, the mean scores of pain intensity differed meaningfully among four groups (*P* = 0.008). (Table 2)

**Table 2:** Pain intensity scores during, just after, and 15 min later after chest tube removal among four groups

Group		During	Just after	15 min later
		Mean ± SD	Mean ± SD	Mean ± SD
Control Group		4.25 ± 0.78	2.85 ± 1.30	1.51 ± 0.65
Respiratory relaxation		4.54 ± 0.7	2.17 ± 1.07	1.51 ± 0.60
Reflexology massage		4.57 ± 0.55	2.14 ± 1.19	1.48 ± 0.52
Combination of respiratory relaxation and reflexology		4.54 ± 0.56	1.45 ± 0.61	1.31 ± 0.67
One-Way ANOVA test	F	1.78	9.83	0.71
	df	3	3	3
	**P value**	**0.15**	**0.001**	**0.54**

For a better understanding of the difference in pain intensity just after chest tube removal among four groups; we used Tukey’s post hoc test. The results showed that all the groups compared to each other were meaningfully different just after chest tube removal, except one pair group which was the reflexology group and respiratory relaxation group (*P* = 0.99).

**Fig. 1:**
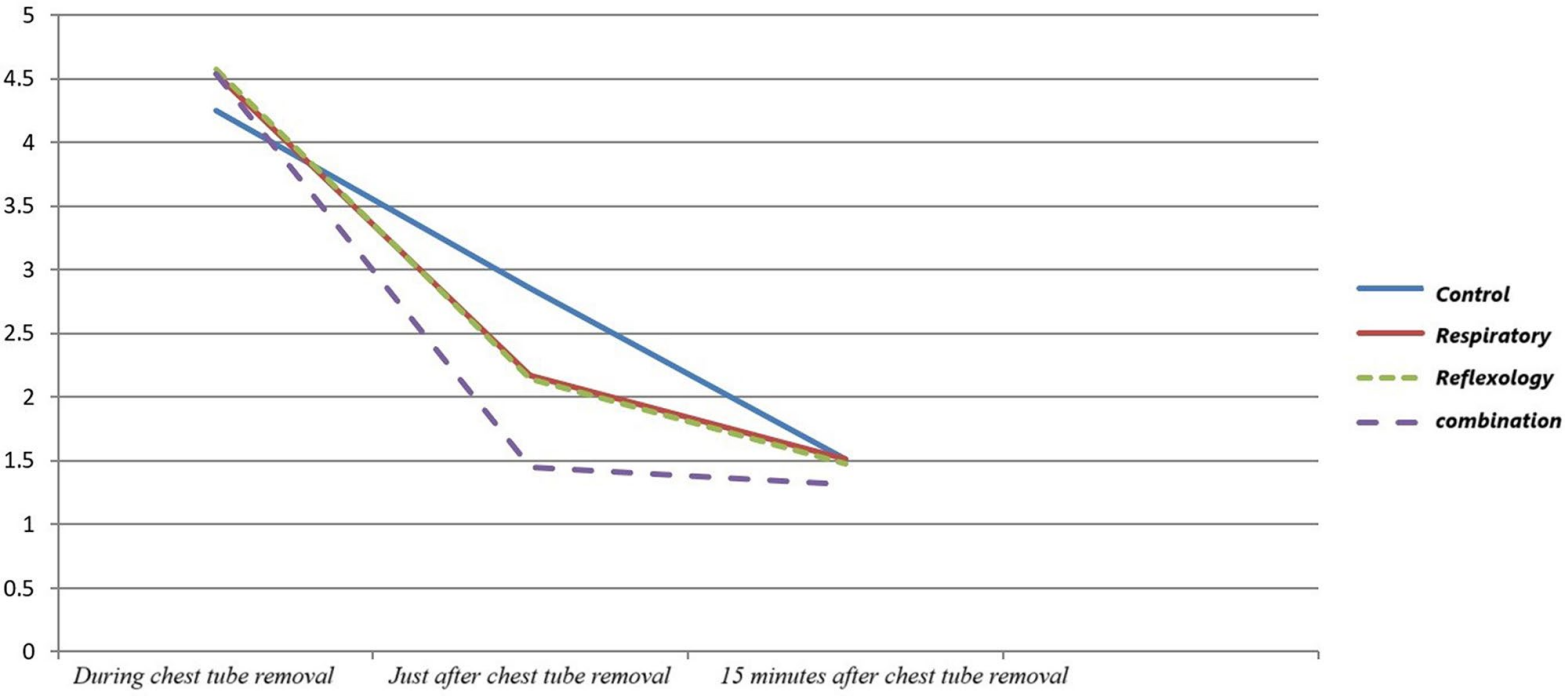
The pain intensity during the study among four groups

The Greenhouse-Geisser correlation test also showed that across all four groups, the pain intensity was meaningfully different in the three timelines in which the pain intensity was assessed (*P* < 0.001). The pain intensity among the four groups during the study is shown in Fig. 1.

## Discussion

The results of this study showed that the average pain intensity immediately after chest tube removal was significantly lower in the intervention groups than in the control group. The combination group in which the respiratory relaxation and foot reflexology were used together had the biggest decrease in terms of pain intensity just after chest tube removal; the control group had the least decrease; and the respiratory relaxation group and foot reflexology group had similar decrease which their effectiveness if they were used alone were almost the same.

In this regard, Goktuna et al. (2023) showed in a study that the mean pain scores in the group that received hand reflexology massage treatment were significantly reduced compared to the placebo group in patients after coronary artery bypass graft surgery [35]. Also, Babatabar et al., in a study conducted in 2018, showed that reflexology massage during chest tube removal after coronary artery bypass surgery is effective in reducing the heart rate and breathing rate back to normal values. This shows that this method may be a beneficial nursing intervention for reducing distress and sympathetic tone [31]. Lee et al., also found foot reflexology massage to be effective in reducing anxiety in patients undergoing coronary artery bypass surgery [43].

Meanwhile, Han and Lee, 2012 reported that massaging of the back did not affect situational anxiety in patients after gastrectomy surgery [44]. It is possible that the result was affected by the small number of samples (29 samples), the type of disease (gastrectomy), cultural-geographical differences (East Asia), or the place of massage (back massage) of the patients.

Considering the high number of open-heart surgeries in the world and since the patient’s anxiety and pain are high during tube removal, the use of foot massage, which is a non-invasive, cost-effective method, can reduce the anxiety and pain of these patients. It can also reduce the use of anti-anxiety drugs [45].

Friesner et al. (2006) showed in a study that a significant reduction in the pain level of patients is observed immediately and 10 min after chest tube removal when a slow deep-breathing relaxation was used alongside the use of opioids, compared to using opioids alone [33]. Also, Ayyasi et al. (2018) in a single-blind trial study that was conducted in 2018 showed that respiratory relaxation is an effective technique for the pain intensity caused by the removal of the chest tube in patients after open heart surgery [11].

Gorji et al. (2014) reported that relaxation methods and the use of cold therapy have relatively equal effects in reducing pain caused by chest tube removal. Although the use of cold can cause an unpleasant feeling in patients, it is less popular among patients [18].

Currently, complementary treatments to increase patient satisfaction, such as respiratory relaxation, are considered one of the effective methods in many parts of the world, and their use is highly recommended for patients. Due to factors such as increasing costs of treating patients and side effects of chemical drugs, finding alternative treatment methods, and providing these services to patients has been the focus of attention [46].

Based on the findings of this study, respiratory relaxation has also a positive effect on the intensity of pain caused by the removal of the chest tube in patients after open heart surgery. Also in our study, no significant difference was found between the effect of reflexology massage and respiratory relaxation was found but the combination of them had more effect than the single use of them.

Respiratory relaxation can reduce stress and lower heart rate and blood pressure levels in open-heart surgery patients. Also, the relaxation of the muscles can reduce the intensity of pain [47, 48]. (Roditi and Robinson, 2011, Hamasaki, 2020). Therefore, in general, reflex massage and respiratory relaxation can be effective in reducing pain and anxiety during chest tube removal after open heart surgery [33, 49].

Reducing pain and improving patient satisfaction is one of the most important parts of nursing care [50]. Patients often are worried about chest tube removal after an open-heart surgery [12]. Some of the evidence-based alternative and complementary therapies can help nurses manage patients better. However, these methods are often unintroduced and missed in clinical settings. Training and awareness of the treatment staff, especially nurses, about the use of reflexology massage and respiratory relaxation and the combination of them to reduce pain following chest tube removal in heart surgery patients can lead to an improvement in the quality of nursing care as well as patient satisfaction.

## Conclusion

The results showed that reflexology massage and respiratory relaxation both reduce pain immediately after chest tube removal in heart surgery patients. Also, the combination of these two techniques and the simultaneous use of them was more effective in reducing the average pain of patients. Therefore, these techniques can be used to reduce the pain of heart surgery patients during chest tube removal.

## Data Availability

No datasets were generated or analysed during the current study.

## Declarations

### Limitations and recommendations

One of the study’s limitations was the limited number of patients in the study setting. For further research, we can propose using multi-center sampling methods in order to reach a bigger sample size. Another limitation was the limited number of similar studies to compare and support the results. Future studies are recommended to investigate other non-pharmacological methods of complementary and alternative medicine on anxiety caused by chest tube removal and, on average pain in other surgeries and with other variables rather than pain.
